# Management of Absent Upper Esophageal Sphincter Opening After Neurological Injury

**DOI:** 10.1002/lary.70630

**Published:** 2026-05-18

**Authors:** Radhika Rawat, Cyrus W. Abrahamson, Kelly Rogers, Jonelyn Langenstein, Jaymie Bromfield, Arjun Seth, James A. Burns, Andrew P. Stein

**Affiliations:** ^1^ Northwestern University Feinberg School of Medicine Chicago Illinois USA; ^2^ Think + Speak Lab, Shirley Ryan AbilityLab Chicago Illinois USA; ^3^ Department of Otolaryngology ‐ Head and Neck Surgery Northwestern Medicine Chicago Illinois USA; ^4^ Department of Neurology Northwestern Medicine Chicago Illinois USA

**Keywords:** cricopharyngeal muscle dysfunction, cricopharyngeal surgery, dysphagia, swallowing therapy, upper esophageal sphincter

## Abstract

**Objectives:**

To evaluate the effects of swallowing therapy and cricopharyngeus (CP) muscle surgery on upper esophageal sphincter (UES) opening, oral intake, and airway protection in patients with absent UES opening following neurological injury.

**Methods:**

Retrospective review of seven patients managed between September 2021 and July 2024 with absent UES opening confirmed by Modified Barium Swallow Study (MBSS). All patients underwent swallowing therapy followed by at least one CP‐targeted intervention, including balloon dilation, botulinum toxin injection, and/or myotomy. Swallowing outcomes were assessed using the Functional Oral Intake Scale (FOIS), Mann Assessment of Swallowing Ability (MASA), Penetration‐Aspiration Scale (PAS), and pharyngoesophageal segment (PES) opening scores from the Modified Barium Swallow Impairment Profile.

**Results:**

CP‐targeted surgeries were associated with improved FOIS, MASA, and PES opening scores. PAS scores did not change substantially, with six patients demonstrating values greater than or equal to 7. Patients who underwent CP‐directed intervention within 6 months of injury demonstrated greater FOIS improvements than those treated later (3.25 vs. 0.33).

**Conclusion:**

CP interventions can facilitate improved bolus passage and oral intake in patients with absent UES opening following neurological injury. However, persistent aspiration risk remains despite improved mechanical opening, highlighting the need for continued swallowing therapy. Finally, earlier intervention may optimize outcomes, though further research is needed to refine treatment for this rare condition.

**Level of Evidence:**

4

## Graphical abstract

This study evaluated the effects of swallowing therapy and cricopharyngeus (CP) muscle surgery on upper esophageal sphincter (UES) opening, oral intake, and airway protection in patients with absent UES opening following neurological injury. CP‐targeted surgeries were associated with improved bolus passage and oral intake, but persistent aspiration risk remained despite improved mechanical opening. These findings highlight the importance of multidisciplinary management of this rare condition and suggest that earlier surgical intervention combined with continued swallowing therapy may optimize recovery following neurological injury.

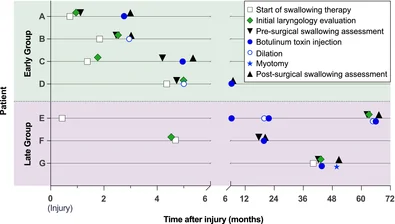

## 1 Introduction

Oropharyngeal dysphagia and cricopharyngeal muscle dysfunction (CPMD) are potential debilitating consequences of neuromuscular disorders and neurological injuries, impairing quality of life and increasing risk of complications such as aspiration pneumonia [1, 2, 3]. The upper esophageal sphincter (UES), primarily composed of the cricopharyngeus (CP) muscle, plays a critical role in coordinated bolus passage from the pharynx to the esophagus [4]. The UES is controlled by a complex neural network involving both cortical and brainstem structures, primarily the nucleus tractus solitarius and dorsal motor nucleus of the vagus, integrating sensory input to coordinate swallowing, including hyolaryngeal elevation and sphincter tone inhibition [5, 6]. Disruptions from cortical stroke, brainstem infarction, or injury to descending motor pathways can result in aberrant or absent UES opening, which can significantly impair swallow function [1, 5].

CPMD is a well‐described form of UES impairment involving incomplete relaxation or hypertonicity due to reduced inhibitory input to the CP [7]. Partial bolus transit is often preserved, and interventions targeting CP tone, including balloon dilation, botulinum toxin (botox) injection, and myotomy, have demonstrated benefit [7, 8, 9, 10]. Efficacy depends on timing relative to neurological injury and extent/location of the underlying injury [11, 12].

By contrast, absent UES opening represents a more severe condition involving complete failure of the UES to open during swallowing with no bolus transit from the hypopharynx into the proximal esophagus. This is often associated with extensive neurological injury, including impaired pharyngeal propulsion, reduced hyolaryngeal excursion, and/or disruption of central pattern generators. Since absent UES opening involves more swallowing components than localized CPMD, it may not respond predictably to swallowing therapy nor surgery.

Indeed, there is minimal information available regarding absent UES opening as a distinct clinical entity, with few studies directly examining its presentation, mechanisms, or treatment response [7]. Therefore, we conducted a retrospective analysis of patients with absent UES opening on Modified Barium Swallow Study (MBSS) following neurological injury to characterize this phenotype and measure outcomes following swallowing therapy and CP‐targeted interventions.

## 2 Methods

### 2.1 Study Design and Patient Selection

This retrospective chart review included all patients managed at Northwestern Memorial Hospital and/or Shirley Ryan AbilityLab from September 2021 through July 2024 with absent UES opening following neurological injury. Inclusion criteria were: documented absent UES opening on MBSS, at least one UES/CP surgery, and neurological insult resulting in dysphagia unresponsive to conventional therapy. This encompassed both acute post‐stroke patients as well as individuals with severe subacute or chronic dysphagia following traumatic brain injury, including those diagnosed with an absent swallow response by a speech‐language pathologist (SLP) on MBSS. Exclusion criteria included structural esophageal abnormalities, cognitive impairment precluding therapy, or contraindications to CP‐targeted surgery. This study was approved by Northwestern's IRB (STU00221862).

### 2.2 Data Collection and Swallowing Assessments

Clinical and demographic data were extracted, including age, sex, etiology of dysphagia, tracheostomy and feeding tube status, time from neurological injury to intervention, and treatment details. Pre‐ and post‐treatment swallowing function were evaluated using MBSS and four validated tools by a SLP: Functional Oral Intake Scale (FOIS), Mann Assessment of Swallowing Ability (MASA), Penetration–Aspiration Scale (PAS), and Component 14 of the Modified Barium Swallow Impairment Profile (MBSImP), which assesses pharyngoesophageal segment (PES) opening (File S1) [13, 14, 15, 16, 17]. In addition to PES opening, other components of the MBSImP were scored pre‐ and post‐treatment, including Component 6 (Initiation of the Pharyngeal Swallow) and components of the Pharyngeal Impairment Domain.

### 2.3 Interventions

All patients received swallowing therapy from an SLP with exercises including a combination of effortful swallow, Mendelsohn maneuver, Masako maneuver, super supraglottic swallow, expiratory muscle strength training, lingual strength training, and chin tuck against resistance (CTAR)/Shaker exercises. Some patients also underwent neuromuscular electrical stimulation (NMES). Vocal fold adduction exercises were not a standard component of treatment, but select patients with unilateral vocal fold paralysis did utilize these when clinically indicated. Therapy occurred in multiple settings and at different frequencies depending on clinical trajectory: acute inpatient rehabilitation (AIR, 4–6 times per week), day rehabilitation (DR, 3–5 times per week), or outpatient (1–3 times per week).

Patients also underwent one or more surgeries, with only one intervention performed per procedure:

- Balloon dilation: Carried out with rigid endoscopic exposure and an 18 mm balloon catheter inflated for 3 min.
- Botox injection: Intramuscular injection of 60 units of botox into the CP muscle under rigid endoscopic guidance.
- Endoscopic CP myotomy.

### 2.4 Outcome Measures and Follow‐Up

The primary outcome included assessment of MBSS‐derived PES opening scores. Secondary measures included changes in FOIS, MASA, and PAS scores as well as the need for repeat intervention. Follow‐up intervals varied based on clinical availability, with outcomes measured via MBSS after CP‐targeted intervention and accompanying clinical swallow evaluations. The duration of benefit following each procedure was assessed based on symptom recurrence and need for repeat surgery.

### 2.5 Statistical Analysis

Descriptive statistics summarized demographic and clinical characteristics. Changes in FOIS, MASA, PES, and PAS (thin liquids) ratings pre‐ versus post‐treatment were analyzed using raw mean differences (Δ). For purposes of outcome analyses, the highest documented post‐treatment FOIS was used. All analyses used GraphPad Prism version 10.

## 3 Results

### 3.1 Patient Characteristics and Swallowing Therapy

The cohort included seven patients with absent UES opening on MBSS following neurological injury (Table 1). Four patients were male, with a mean age of 43.0 ± 13.5 years. Five patients had a tracheostomy at some point during their disease course. All patients were unsafe to eat by mouth and dependent on enteral nutrition. Underlying neurological injury, as outlined in Table 2, was unique for each patient, including a wide range of issues such as cerebellar hemorrhage, traumatic brainstem stroke after high‐speed snowmobile accident, and lightning strike causing cardiac arrest.

**TABLE 1:** Demographic characteristics and comorbidities of the cohort. Age, sex, and medical comorbidities reflect status at the time of neurological injury.

Characteristic	All Patients (*N* = 7)
Age (mean ± SD) at injury	43 ± 13.5
20–44 years	5 (71%)
45–64 years	1 (14%)
65+ years	1 (14%)
Sex	
Female	3 (43%)
Male	4 (57%)
Race (self‐identified)	
White	5 (71%)
Black or African American	1 (14%)
Other/Unknown	1 (14%)
Medical Comorbidities	
Hypertension	2 (29%)
Hyperlipidemia	1 (14%)
Migraines	2 (29%)
Prior Stroke or Transient Ischemic Attack	1 (14%)
Obstructive Sleep Apnea	1 (14%)
Etiology of Neurological Injury	
Brainstem infarct	2 (29%)
Medullary cavernoma with hemorrhage	1 (14%)
Posterior fossa arteriovenous malformation rupture	1 (14%)
Anoxic brain injury	1 (14%)
Cerebellar hemorrhage	1 (14%)
Traumatic cranial neuropathy	1 (14%)
Tracheostomy status^a^	
Current	3 (43%)
Prior	2 (29%)
None	2 (29%)

^a^ Tracheostomy status reflects condition at the time of initial laryngology evaluation.

**TABLE 2:** Brief clinical history, time to swallowing therapy, and duration of treatment during acute inpatient rehabilitation (AIR), day rehabilitation (DR), and outpatient care by patient.

Patient	Brief Clinical History and Neurologic Injury	Time to Initiation of Swallow Therapy (Days (Months))	AIR Duration (Days)	DR Duration (Days)	Outpatient Duration (Days)
A	Cerebellar hemorrhage after craniotomy for epidermoid cyst resection	22 (0.7)	48	296	129
B	Middle cerebral artery territory cortical and subcortical infarcts	64 (2.1)	49	286	0^a^
C	Traumatic brainstem stroke following high‐speed snowmobile accident	42 (1.4)	57	55	0^a^
D	Medullary cavernoma with hemorrhage	133 (4.4)	27	0^a^	0^a^
E	Posterior circulation infarcts from right vertebral artery dissection	13 (0.4)	52	156	0^a^
F	Right cerebellar hemorrhage and brainstem compression from arteriovenous malformation rupture	121 (4.0)	31	114	0^a^
G	Lightning strike leading to cardiac arrest and anoxic brain injury	1224 (40.5)	134	0^a^	0^a^

^a^ Duration of 0 indicates no documented therapy.

**FIGURE 1:**
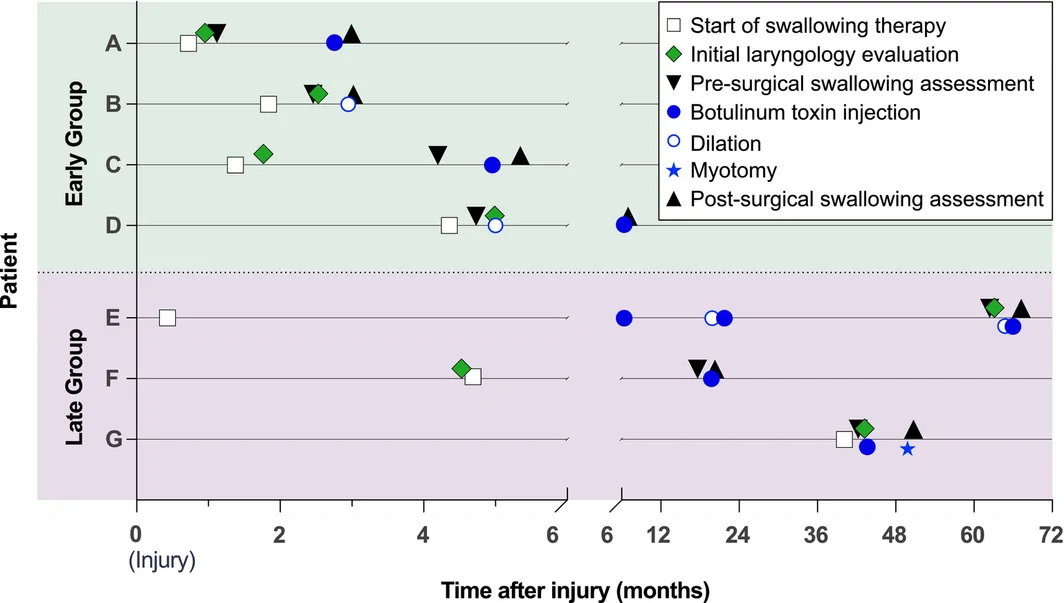
Clinical timelines of interventions and swallowing assessments for individual patients. Symbols indicate the timing of key clinical events for each patient. Note: X‐axis is discontinuous and unequally scaled to show all relevant events. [Color figure can be viewed in the online issue, which is available at www.laryngoscope.com]

Time from neurological injury to start of swallowing therapy was variable (Table 2 and Figure 1). Patient G presented from abroad for additional swallowing rehabilitation more than 3 years after initial injury. Therefore, therapy timing was evaluated with and without Patient G. Mean time from injury to swallowing therapy initiation for all patients was 7.7 ± 14.4 months compared to 2.2 ± 1.9 months when excluding Patient G. Mean AIR duration was 56.9 ± 35.8 days (*n* = 7), while DR averaged 181.4 ± 106.3 days (*n* = 5). Only one patient underwent outpatient therapy (129 days).

During their course of therapy and prior to considering surgery, all patients underwent MBSS and clinical swallow evaluation (Table 3). PES scores were all 3, consistent with absent UES opening. Mean FOIS scores were 1.14 ± 0.38, reflecting complete or near‐complete enteral dependence. Average MASA scores were 131.3 ± 37.0, all below thresholds considered safe for oral intake. Finally, mean PAS scores were 7.57 ± 0.53, indicating significant aspiration risk.

**TABLE 3:** Patient outcomes following cricopharyngeal‐targeted interventions.

Patient	Pre PES	Post PES	Pre FOIS	Post FOIS	Pre MASA	Post MASA	Pre PAS	Post PAS	Time from Final Surgical Intervention to MBSS (days)	Outcome
A	3	2	1	5	140	^a^	7	8	7	Oral intake, PEG tube retained
B	3	1	1	7	167	184	7	6	15	Full oral intake (no PEG tube)
C	3	1	1	2	92	^a^	8	7	12	Initiated trials, partial oral intake, some progress
D	3	1	1	3	164	171	7	8	19	Partial improvement, later declined due to medical complications
E	3	3	2	1	161	^a^	8	8	9	No improvement
F	3	1	1	2	121	162	8	8	9	Minimal improvement
G	3	2	1	2	74	99	8	7	27	PEG tube dependent, PO intake trials

*Note:* Light gray shading indicates the early‐treated group (Patients A–D) while Patients E–G represent the late‐treated cohort.

Abbreviations: FOIS, Functional Oral Intake Scale; MASA, Mann Assessment of Swallowing Ability; MBSS, Modified Barium Swallow Study; PAS, Penetration–Aspiration Scale; PEG, Percutaneous Endoscopic Gastrostomy; PES, Pharyngoesophageal Segment opening from Modified Barium Swallow Impairment Profile; PO, By mouth.

^a^ No post‐treatment score available due to transfer of care, loss to follow‐up, or death.

### 3.2 Surgical Interventions

All patients were referred for surgical consideration due to the severity of their swallowing impairment in the setting of absent UES opening. Laryngology evaluation demonstrated vocal fold immobility in six patients (85.7%), most commonly unilateral paralysis (*n* = 4, 57.1%) (Table 4). Soft palate weakness was present in three patients (42.9%). These findings were consistent with neurological insult involving high vagal pathways, likely contributing to absent UES opening.

**TABLE 4:** Laryngoscopy findings and cricopharyngeal‐targeted interventions.

Patient	Vocal Fold Paralysis?	Soft Palate Weakness?	Time from Injury to Surgical Intervention (months)	Surgical Intervention(s)
A	Right	Yes	2.8	Botox injection (1×)
B	Left	No	3.0	Dilation (1×)
C	Bilateral	Yes	5.0	Botox injection (1×)
D	Bilateral	No	5.0	Dilation (1×), Botox injection (1×)
E	Right	No	6.3/64.9^a^	Dilation (2×), Botox injection (3×)
F	Normal mobility	Yes	19.9	Botox injection (1×)
G	Left	No	43.7	Botox injection (1×), Myotomy (1×)

Abbreviation: Botox, botulinum toxin.

^a^ Patient E underwent initial evaluation and surgical interventions at another institution just over 6 months after injury before presenting for care with us nearly 5 years later.

Mean time from injury to first CP‐directed surgery for all patients was 12.2 ± 15.1 months. When excluding Patient G, the average time to first procedure was 7.0 ± 6.5 months. Four patients completed their initial surgery within 6 months of injury (early‐treated), while three underwent surgery more than 6 months later (late‐treated) [15, 18]. Patient E was originally managed at an outside institution just over 6 months after initial injury, and subsequently presented to our team 5 years later. Their first surgery with us was 64.9 months post‐injury. Initial surgeries were CP balloon dilation for two patients and botox injection for the other five. Three patients had multiple surgeries: Patients D, G, and E (Table 4 and Figure 1).

### 3.3 Functional Swallowing Outcomes

#### 3.3.1 PES Opening Ratings

**FIGURE 2:**
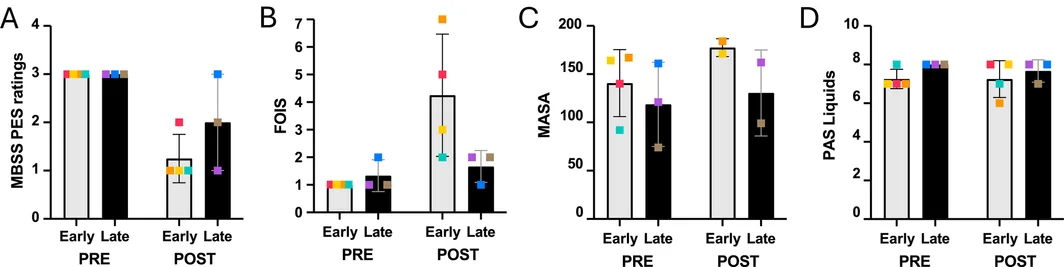
Functional and physiological swallowing outcomes. Colored dots represent individual patients: magenta (A), orange (B), teal (C), yellow (Patient D), blue (E), purple (F), and brown (G). Error bars represent standard deviations. PES, Pharyngoesophageal Segment opening; MBSS, Modified Barium Swallow Study; FOIS, Functional Oral Intake Scale; MASA, Mann Assessment of Swallowing Ability; PAS, Penetration–Aspiration Scale; PEG, Percutaneous Endoscopic Gastrostomy; PO, By Mouth. [Color figure can be viewed in the online issue, which is available at www.laryngoscope.com]

PES opening improved from 3.00 ± 0.00 pre‐treatment to 1.57 ± 0.79 post‐procedure, indicating increased bolus passage through the UES (*n* = 7, Δ = −1.43) (Table 3 and Figure 2A). Mean PES opening scores decreased by 1.75 ± 0.5 in the early‐treated group, while they declined by a mean of 1 ± 1 in the late‐treated group.

#### 3.3.2 FOIS Scores

Mean FOIS scores increased from 1.14 ± 0.38 to 3.14 ± 2.12, reflecting tube dependence with consistent oral intake of liquids and food following CP‐targeted interventions (*n* = 7, Δ = 2.00) (Table 3 and Figure 2B). Among early‐treated patients, the mean FOIS score increased by 3.25 ± 2.22 points, compared to 0.33 ± 1.15 points in late‐treated patients. In categorical analysis, 2 of 4 early‐treated patients (50%) achieved a FOIS score ≥ 4, compared to 0 late‐treated patients.

#### 3.3.3 MASA Scores

Mean MASA scores increased from 131.29 ± 37.05 pre‐treatment (*n* = 7) to 154.00 ± 37.76 post‐treatment (*n* = 4), indicating continued dysphagia with borderline moderate impairment and aspiration risk. However, only four patients (B, D, F, and G) had both pre‐ and post‐treatment MASA scores available for evaluation. The average MASA increase was 22.50 ± 14.36 for these four patients (Table 3 and Figure 2C). MASA scores rose 12.00 ± 7.07 points in the early‐treated group and 33 ± 11.31 points in the late‐treated group.

#### 3.3.4 PAS Scores

PAS scores for thin liquids remained high, with a mean of 7.57 ± 0.53 pre‐treatment and 7.43 ± 0.79 post‐surgery (*n* = 7, Δ = −0.14) (Table 3 and Figure 2D). Mean PAS scores in the early‐treated group decreased by 0 ± 0.96, and in the late‐treated group by 0.33 ± 0.58. Six patients (85.7%) had post‐treatment PAS scores ≥ 7, indicating that they remained at high risk for aspiration.

#### 3.3.5 Initiation of the Pharyngeal Swallow and Pharyngeal Impairment Domain Scores

The additional MBSImP scores aside from the PES opening score revealed issues across all components of the pharyngeal swallow for this cohort (Table S1). Initiation of the pharyngeal swallow varied substantially across patients at baseline, with Patients E and F showing no visible initiation of the pharyngeal swallow at any location. Late‐treated patients had worse scoring on the initiation of the pharyngeal swallow compared to early‐treated patients (3.67 ± 0.58 vs. 1.75 ± 1.50), and scoring was unchanged following surgery except in Patient F.

Similarly, following surgery some patients showed modest improvements in individual components in the Pharyngeal Impairment Domain, though impairments largely persisted, particularly with laryngeal vestibule closure and tongue base retraction. Scores between the early‐ and late‐treated groups were similar before and after procedural intervention, and neither group appeared to demonstrate a greater improvement in the pharyngeal swallow.

#### 3.3.6 Clinical Course

Follow‐up duration ranged from 1 to 30 months post‐intervention for all patients. Patient B discontinued gastrostomy tube feeds during follow‐up and maintained full oral intake (FOIS score 7) for at least 9 months. Patient A achieved a FOIS score of 5, indicating oral intake of multiple consistencies with special preparation, but remained partially gastrostomy tube dependent. Patient C advanced to FOIS 2 after initiating thin liquid trials following botox injection; however, continued tube dependence was noted at last follow‐up. The remaining patients (D, E, F, and G) continued to require partial or exclusive enteral nutrition, with minimal to no documented improvement in oral intake.

Long‐term outpatient documentation was limited. Patients B and G were discharged with home exercise programs but did not return for structured outpatient sessions. Patient D passed away shortly after discharge from unrelated medical causes. Patient C transferred care to an outside institution and post‐intervention records were incomplete.

## 4 Discussion

Absent UES opening represents a more profound disruption in neural initiation and swallowing coordination as compared to the more commonly described clinical condition of CPMD. While prior studies have described benefits of botox injection, dilation, and myotomy for CPMD, they have rarely addressed absent UES opening as a distinct condition [7, 9, 10, 19]. In this manner, there is limited guidance for the management or prognosis of patients with this unique clinical entity [7, 19]. Therefore, we evaluated functional outcomes following swallowing therapy and CP‐directed interventions in a cohort of seven patients with absent UES opening and gastrostomy tube dependence after neurological injury. To our knowledge, this represents the largest series of patients described in the literature who were managed for this rare condition.

Six patients began swallowing therapy within 6 months of their initial neurological injury. One patient (Patient G) started therapy over 3 years post‐injury, which was likely related to the severity of his initial injury (lightning strike leading to cardiac arrest and anoxic brain injury) and inability to participate in therapy earlier in his course. Since patients with absent UES opening do not have any bolus passage into the proximal esophagus, it was not surprising to see that none of the patients in this study progressed their diet with therapy alone. However, we feel it is important to engage in therapy as soon as feasible since sustained rehabilitation may preserve pharyngeal coordination and optimize responsiveness to surgery.

Following CP‐targeted procedures, there was improved bolus passage through the UES for six of the seven patients as demonstrated by a decrease in their PES opening rating. Additionally, overall swallowing ability improved as reflected by increases in FOIS and MASA scores. Specifically, all six patients with enhanced PES opening on post‐treatment MBSS also demonstrated increased FOIS scores. Patient B showed the most marked improvement, progressing from FOIS 1 pre‐intervention to 7 (full oral intake) within 2 weeks post‐surgery. Although we noted global progress in swallow function post‐surgery, six patients had post‐treatment PAS scores ≥ 7, indicating persistent aspiration risk. Thus, while CP‐directed interventions improved some functional outcomes, they did not consistently improve airway protection.

One explanation for these findings is the MBSImP Pharyngeal Impairment Domain data, which demonstrated that while PES opening improved following CP‐targeted intervention, deficits in other key motor components of the pharyngeal swallow persisted. Particularly, impaired tongue base retraction to the posterior pharyngeal wall reduces the driving pressure available for bolus propulsion, limiting the functional benefit of improved UES opening [19, 20]. Additionally, poor hyolaryngeal elevation and anterior hyoid excursion further compromise the biomechanical forces needed for both UES opening and airway protection, as laryngeal excursion contributes directly to cricoid cartilage tipping, UES stretch opening, and laryngeal vestibule closure [4, 20, 21, 22]. Incomplete laryngeal vestibule closure, present in several patients, further elevates aspiration risk [22, 23]. Impaired initiation of the pharyngeal swallow further underscores the centrally mediated nature of this condition as CP‐targeted interventions address only a distal mechanical component of a more globally impaired swallow sequence for patients with no visible swallow initiation at any bolus location. Thus, the persistence of upstream pharyngeal motor deficits likely explains why many patients continued to aspirate even though they demonstrated improved bolus passage through the UES.

Together, the PAS and pharyngeal swallow findings suggest that despite enhanced mechanical opening, global impairments in neuromuscular coordination, including airway protection and pharyngeal timing, persisted. This highlights that CP‐targeted procedures may address only one component of a multistep, neurally coordinated sequence and that gains at the UES level may be functionally limited when propulsive and protective mechanisms remain impaired. These findings also provide a rationale for adjunctive neuromuscular approaches, including NMES, that may augment hyolaryngeal muscles, though their efficacy in severe, centrally mediated dysphagia requires further investigation.

Notably, patients who underwent CP‐directed procedures within 6 months (early‐treated) showed greater gains in swallowing function and oral intake than those treated after 6 months (late‐treated). This was demonstrated by the early‐treated group having a nearly 1 point greater improvement in PES opening rating and a 3‐point increase in FOIS scores compared to the late‐treated patients. Additionally, two early‐treated patients reached FOIS scores ≥ 4, compared to none of the late‐treated patients. This suggests earlier intervention may mitigate disuse atrophy, maladaptive neural reorganization, and pharyngeal deconditioning. This aligns with prior findings in post‐stroke dysphagia, where delayed restoration of oral intake has been linked to worsened pharyngeal function and reduced neuroplasticity [1, 19].

Differences in clinical histories likely contributed to variability in treatment response as well. Two late‐treated patients showed poor response despite multiple interventions. Patient E had a posterior circulation stroke over 5 years prior and previously underwent dilation and two botox injections at outside institutions without benefit. Following repeat CP interventions with our team, PES opening, PAS score, and FOIS did not improve. Similarly, Patient G, with longstanding anoxic brain injury and global neurological dysfunction, underwent CP botox injection and subsequently endoscopic myotomy with only modest improvement in PES opening and no durable clinical recovery or functional gains. These cases suggest that longstanding disuse, extensive central injury, and previous nonresponse to surgery may signal poor prognosis for functional swallowing recovery.

In contrast, Patient B demonstrated the most favorable recovery. They initiated swallowing therapy within 2 months of their stroke and had CP dilation within 3 months. Notably, preoperative clinical indicators suggested less severe neuromuscular impairment, including a high pre‐treatment MASA score of 167 (threshold = 170), which may have contributed to their rapid improvement after surgery. Unlike others, they achieved full oral intake without dietary restrictions (FOIS 7) and discontinued all enteral support. This suggests that the degree of cortical injury, early swallowing therapy, and prompt surgery may contribute synergistically to a more robust recovery. Indeed, the observed variability in treatment response underscores the importance of individualized management.

Finally, a distinctive feature of this cohort is the inclusion of patients with functionally absent swallow response. For these individuals, interventions were not aimed at restoring typical swallow physiology but rather at optimizing safety and enhancing oral intake for quality‐of‐life purposes, such as pleasure feeds. This broadens the clinical relevance of our findings, suggesting a potential role for targeted interventions even in patients with severe chronic dysphagia who fall outside the traditional post‐stroke populations.

Several limitations must be acknowledged, including small sample size, retrospective design, and heterogeneity in both neurologic injury and intervention timing. Given the small cohort size, this study was not powered to detect subtle differences between treatment groups or intervention modalities, and formal statistical analyses could not be performed. Variability in swallowing therapy modalities and follow‐up intervals also limited our ability to assess dose–response relationships. In addition, the absence of high‐resolution manometry precluded direct assessment of pharyngeal pressures and CP tone, which may have clarified the pathophysiological basis of each patient's underlying CP dysfunction and response to treatment. These limitations underscore the need for larger, prospective studies incorporating objective physiologic measures and standardized therapeutic protocols to understand treatment options for patients with this rare clinical issue.

Overall, there is limited information on the management of patients with absent UES opening, in particular the success of different surgical interventions directed at the CP muscle. This work suggests benefit of different CP‐targeted interventions in patients with absent UES opening and that earlier surgical intervention may be beneficial. However, the variable response between patients reinforces that absent UES opening likely reflects central pattern generator deficit, and that CP‐targeted interventions alone may not overcome broader impairments in swallow initiation, pharyngeal propulsion, hyolaryngeal excursion, and/or airway closure. In this manner, swallowing therapy pre‐ and post‐surgery remains critical for these patients' recovery.

## 5 Conclusions

This study details the management of the largest series of patients with absent UES opening described in the literature. No patients advanced beyond FOIS 2 prior to surgery, suggesting that therapy alone is insufficient in cases of absent UES opening. Moreover, the trend toward better outcomes with earlier surgery suggests that sooner laryngology referral and intervention may enhance a patient's clinical trajectory for swallowing rehabilitation. Continued therapy post‐procedure also likely contributes to gains in bolus tolerance and maintenance of function. Overall, the findings of this work emphasize the importance of multidisciplinary management of patients with severe dysphagia and gastrostomy tube dependence due to absent UES opening following neurological injury. Future work should examine the interaction between swallowing therapy intensity, timing of surgery, and neuromuscular phenotypes to refine rehabilitation strategies for this complex patient population.

## Funding

The authors have nothing to report.

## Conflicts of Interest

The authors declare no conflicts of interest.

## Supporting information


**File S1:** Swallowing Assessment Scores.

**Table S1:** Pharyngeal swallow evaluation and scoring prior to and following cricopharyngeal‐targeted interventions using MBSImP. Light gray shading indicates the early‐treated group (patients A–D) while patients E–G represent the late‐treated cohort. MBSImP, Modified Barium Swallow Impairment Profile.

## Data Availability

The data that support the findings of this study are available from the corresponding author upon reasonable request.
