# Supplementary material for: Management of Absent Upper Esophageal Sphincter Opening After Neurological Injury
Source: Laryngoscope. 2026 May 18;136(10):4383–91. doi: 10.1002/lary.70630 (PMC13569688; doi:10.1002/lary.70630)
Supplement: Supplementary file 1 [file LARY-136-4383-s001.docx]

| **MBSImP^TM^ Component** | **Pre A** | **PostA** | **Pre B** | **Post B** | **Pre C** | **Post C** | **Pre D** | **Post D** | **Pre E** | **Post E** | **Pre F** | **Post F** | **Pre G** | **Post G** |
| --- | --- | --- | --- | --- | --- | --- | --- | --- | --- | --- | --- | --- | --- | --- |
| Initiation of the Pharyngeal Swallow | 3 | 3 | 1 | 1 | 3 | 3 | 0 | 0 | 4 | 4 | 4 | 3 | 3 | 3 |
| Laryngeal Elevation | 1 | 2 | 1 | 1 | 3 | 3 | 1 | 0 | 3 | 3 | 2 | 1 | 1 | 1 |
| Anterior Hyoid Excursion | 2 | 1 | 1 | 1 | 2 | 2 | 2 | 2 | 2 | 2 | 1 | 2 | 2 | 1 |
| Epiglottis Movement | 2 | 1 | 2 | 1 | 2 | 2 | 1 | 1 | 2 | 2 | 1 | 2 | 2 | 2 |
| Laryngeal Vestibule Closure | 1 | 2 | 1 | 1 | 2 | 2 | 1 | 2 | 2 | 2 | 2 | 2 | 1 | 1 |
| Pharyngeal Stripping Wave | 2 | 1 | 2 | 2 | 2 | 2 | 1 | 1 | 2 | 2 | 2 | 1 | 1 | 2 |
| Pharyngoesophageal Segment Opening | 3 | 2 | 3 | 1 | 3 | 1 | 3 | 1 | 3 | 3 | 3 | 1 | 3 | 2 |
| Tongue Base Retraction | 3 | 3 | 0 | 0 | 3 | 3 | 3 | 2 | 3 | 3 | 3 | 3 | 2 | 3 |
| Pharyngeal Residue | 4 | 3 | 4 | 3 | 4 | 4 | 3 | 3 | 4 | 4 | 4 | 2 | 3 | 2 |

Supplemental Table 1: Pharyngeal swallow evaluation and scoring prior to and following cricopharyngeal-targeted interventions using MBSImP. Light gray shading indicates the early-treated group (patients A-D) while patients E-G represent the late-treated cohort.

*MBSImP^TM^: Modified Barium Swallow Impairment Profile*
