# Supplementary material for: Management of Absent Upper Esophageal Sphincter Opening After Neurological Injury
Source: Laryngoscope. 2026 May 18;136(10):4383–91. doi: 10.1002/lary.70630 (PMC13569688; doi:10.1002/lary.70630)
Supplement: Supplementary file 2 [file LARY-136-4383-s002.docx]

**Supplemental File 1: Swallowing Assessment Scores**

Pharyngoesophageal Segment (PES) opening was assessed using Component 14 of the MBSImP protocol, which evaluates three dimensions: distention (width of segment opening), duration (time open), and obstruction to bolus flow.^1^ For the purposes of this study, we use “UES” when discussing anatomical and surgical aspects, and “PES” when referring to scoring conventions within the MBSImP. Scores are based on the frame of maximal PES distention during swallowing.

| Score 0 | Straight PES borders, no narrowing, complete bolus flow |
| --- | --- |
| Score 1 | Partial distention or duration with some obstruction (e.g., narrowing, early collapse, or reduced anterior hyoid excursion) |
| Score 2 | Minimal distention and duration with marked resistance due to narrowing, rapid collapse, or both |
| Score 3 | Complete absence of PES opening with no bolus clearance |

The Functional Oral Intake Scale (FOIS) is a 7-point ordinal scale that describes a patient's level of oral intake.^2^

| Score 1 | Nothing by mouth (NPO) |
| --- | --- |
| Score 2 | Tube dependent with minimal/inconsistent oral intake |
| Score 3 | Tube dependent with consistent oral intake of liquids/food |
| Score 4 | Total oral intake of a single consistency |
| Score 5 | Total oral intake with multiple consistencies, but requiring special preparation or compensation |
| Score 6 | Total oral intake with no special preparation, but with specific food limitations |
| Score 7 | Total oral intake with no restrictions |

The Mann Assessment of Swallowing Ability (MASA) evaluates global swallowing function on a 200-point scale.^3,4^

Scores ≤170: dysphagia

Scores ≤150: moderate-to-severe impairment and increased aspiration risk

The Penetration–Aspiration Scale (PAS) is an 8-point scale that quantifies the depth and response to airway invasion observed on MBSS.^5^ Scores ≥7 were considered indicative of significant aspiration.

| Score 1 | Material does not enter the airway |
| --- | --- |
| Score 2 | Material enters the airway, remains above the vocal folds, and is ejected |
| Score 3 | Material enters the airway, remains above the vocal folds, and is not ejected |
| Score 4 | Material contacts the vocal folds, and is ejected |
| Score 5 | Material contacts the vocal folds, and is not ejected |
| Score 6 | Material passes below the vocal folds, and is ejected |
| Score 7 | Material passes below the vocal folds, and is not ejected |
| Score 8 | Material passes below the vocal folds, and no effort is made to eject (silent aspiration) |

In addition to PES opening, the broader pharyngeal motor response was also scored using the MBSImP.^1^ These components evaluate the biomechanical events preceding and facilitating PES opening, including the initiation of the pharyngeal swallow, laryngeal elevation, anterior hyoid excursion, epiglottis movement, laryngeal vestibule closure, pharyngeal stripping wave, tongue base retraction, and pharyngeal residue. All pharyngeal components were scored on lateral view per standard MBSImP protocol.

Initiation of the Pharyngeal Swallow

| Score 0 | Bolus head at posterior angle of ramus (first hyoid excursion) |
| --- | --- |
| Score 1 | Bolus head in valleculae |
| Score 2 | Bolus head at posterior laryngeal surface of epiglottis |
| Score 3 | Bolus head in pyriforms |
| Score 4 | No visible initiation at any location |

Laryngeal Elevation

| Score 0 | Complete superior movement of thyroid cartilage with complete approximation of arytenoids to epiglottic petiole |
| --- | --- |
| Score 1 | Partial superior movement of thyroid cartilage/partial approximation of arytenoids to epiglottic petiole |
| Score 2 | Minimal superior movement of thyroid cartilage with minimal approximation of arytenoids to epiglottic petiole |
| Score 3 | No superior movement of thyroid cartilage |

Anterior Hyoid Excursion

| Score 0 | Complete anterior movement |
| --- | --- |
| Score 1 | Partial anterior movement |
| Score 2 | No anterior movement |

Epiglottis Movement

| Score 0 | Complete inversion |
| --- | --- |
| Score 1 | Partial inversion |
| Score 2 | No inversion |

Laryngeal Vestibule Closure

| Score 0 | Complete; no air/contrast in laryngeal vestibule |
| --- | --- |
| Score 1 | Incomplete; narrow column air/contrast in laryngeal vestibule |
| Score 2 | None; wide column of air/contrast in laryngeal vestibule |

Pharyngeal Stripping Wave

| Score 0 | Present – complete |
| --- | --- |
| Score 1 | Present – diminished |
| Score 2 | Absent |

Tongue Base Retraction

| Score 0 | No contrast between TB and posterior pharyngeal wall (PW) |
| --- | --- |
| Score 1 | Trace column of contrast or air between TB and PW |
| Score 2 | Narrow column of contrast or air between TB and PW |
| Score 3 | Wide column of contrast or air between TB and PW |
| Score 4 | No visible posterior motion of TB |

Pharyngeal Residue

| Score 0 | Complete pharyngeal clearance |
| --- | --- |
| Score 1 | Trace residue within or on pharyngeal structures |
| Score 2 | Collection of residue within or on pharyngeal structures |
| Score 3 | Majority of contrast within or on pharyngeal structures |
| Score 4 | Minimal to no pharyngeal clearance |

**References**

1.	Martin-Harris B, Brodsky MB, Michel Y, et al. MBS measurement tool for swallow impairment--MBSImp: establishing a standard. *Dysphagia*. Dec 2008;23(4):392-405. doi:10.1007/s00455-008-9185-9

2.	Crary MA, Mann GD, Groher ME. Initial psychometric assessment of a functional oral intake scale for dysphagia in stroke patients. *Arch Phys Med Rehabil*. Aug 2005;86(8):1516-20. doi:10.1016/j.apmr.2004.11.049

3.	Mann G. *MASA, the Mann assessment of swallowing ability*. Clifton Park, NY : Singular Thomson Learning; 2002.

4.	Mann G, Hankey GJ, Cameron D. Swallowing function after stroke: prognosis and prognostic factors at 6 months. *Stroke*. Apr 1999;30(4):744-8. doi:10.1161/01.str.30.4.744

5.	Rosenbek JC, Robbins JA, Roecker EB, Coyle JL, Wood JL. A penetration-aspiration scale. *Dysphagia*. Spring 1996;11(2):93-8. doi:10.1007/bf00417897
